# A case of pregnancy and lactation associated osteoporosis in the third pregnancy; robust response to teriparatide despite delayed administration

**DOI:** 10.1016/j.bonr.2020.100706

**Published:** 2020-08-13

**Authors:** Ethem Turgay Cerit, Mahinur Cerit

**Affiliations:** a Ankara Memorial Hospital, Endocrinology and Metabolism Department, Ankara, Turkey; b Gazi University Faculty of Medicine, Radiology Department, Ankara, Turkey

**Keywords:** Pregnancy, Lactation, Osteoporosis, Parathyroid hormone related peptide

## Abstract

**Background:**

Pregnancy and lactation associated osteoporosis (PLO) is a rare condition that may present with fragility fractures occurring for the first time in pregnancy or postpartum period while breastfeeding. Here, we report a rare case of PLO in the 3rd pregnancy treated with teriparatide.

**Case report:**

A 35-year-old woman who presented with back pain (visual analogue scale; VAS = 10/10) two months after her third delivery. PLO was diagnosed from multiple vertebral fragility fractures and low bone mineral density (BMD). She was treated with teriparatide and her pain significantly reduced in the second month. After 12 months of teriparatide treatment, her BMD increased 18.1% from the baseline.

**Conclusion:**

PLO should be considered in patients who complain with back pain during late pregnancy and postpartum period. Weaning off breastfeeding and supplementation of calcium/vitamin D should be the first recommendation as conventional treatment after the diagnosis of PLO. Teriparatide may be an effective option to improve the recovery of BMD If there is not enough improvement with conventional treatment.

## 1 Introduction

Pregnancy and lactation associated osteoporosis (PLO) is a rare type of osteoporosis presenting with vertebral compression fractures and back pain. PLO first appears during the third trimester of pregnancy or in early postpartum period during lactation. It was firstly reported by Nordin and Roper in 1955 (Nordin and Roper, 1955; Kovacs and Ralston, 2015). The pathogenesis of PLO is unclear and etiology is uncertain. Since PLO patients are premenopausal young patients, basal bone mineral density (BMD) values are generally absent and it is not possible to distinguish whether PLO is on the background of osteopenia or on the background of osteoporosis. There are no accepted diagnostic criteria. PLO should be suspected in pregnant or breastfeeding woman with severe, persistent back and/or lower back pain. In the suspected case, after eliminating the causes of secondary osteoporosis, PLO is diagnosed by showing compression fractures with vertebra magnetic resonance imaging (MRI) and measuring BMD with dual energy x ray absorptiometry (DXA) (Kovacs and Ralston, 2015).

Here, we describe a case of PLO after the third pregnancy treated with teriparatide (rhPTH 1–34) and then review the literature about PLO.

## 2 Case presentation

**Fig. 1:**
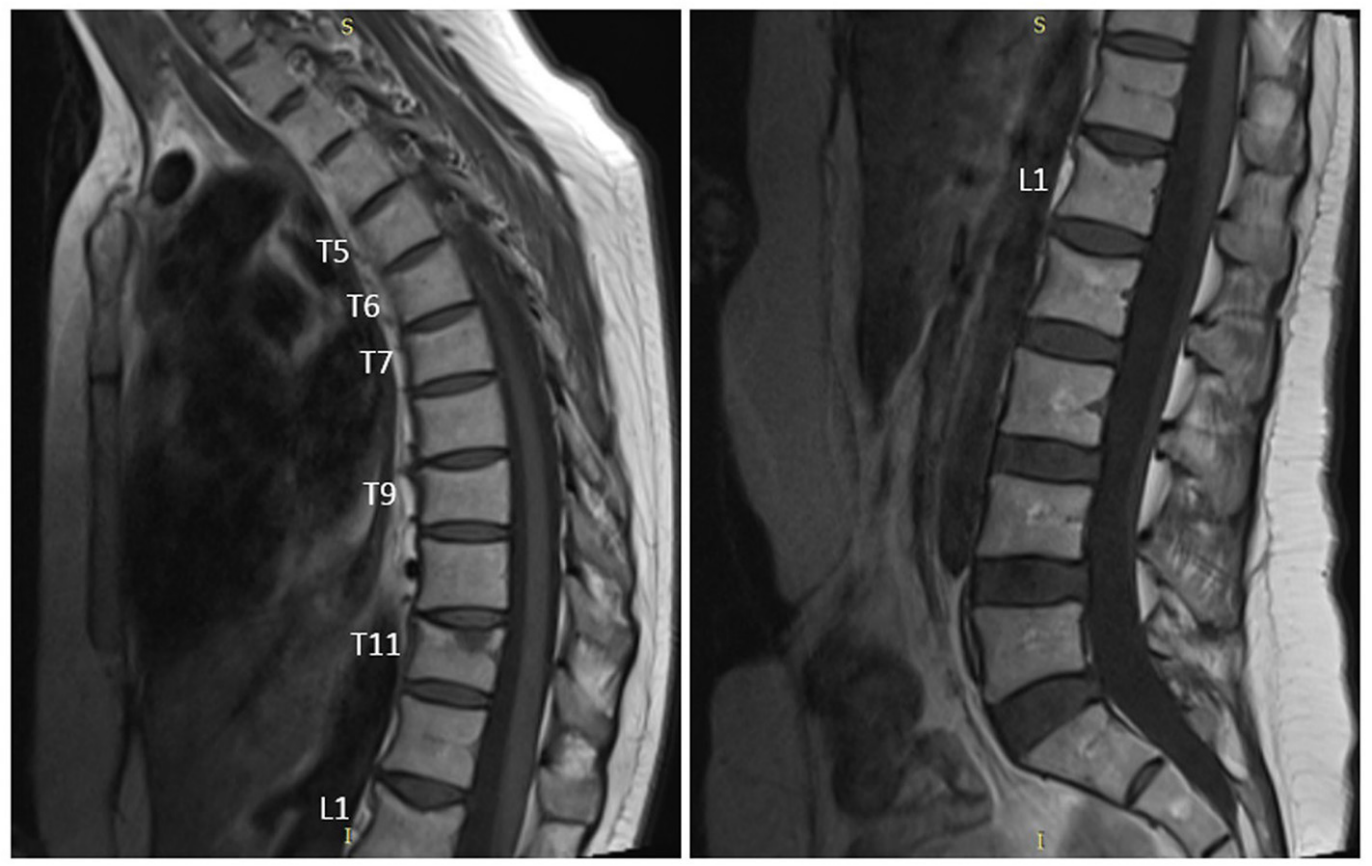
Sagittal T1 magnetic resonance images of the thoracic and lumbar spine shows acute bone marrow edema at T11 vertebra and loss of height at T5–7, 9 and L1 vertebrae (height loss ratios at T5, T6, T7, T9 and L1 are 24%, 25%, 23%, 24%, 27% respectively).

A 35-year-old woman was admitted with severe back pain (visual analogue scale; VAS = 10/10) after her 3rd pregnancy. Acute bone marrow edema was observed at the level of T11 vertebra and loss of height was observed at T5–7, 9 and L1 vertebrae in the thoracolumbar MRI (Fig. 1). Her family had no history of osteoporotic fractures. There was no history of using heparin or any other medication at risk for osteoporosis. She did not smoke and had no history of alcohol use. She had a sedentary adolescence. There was no history of using oral contraceptives. She had breastfeeding for 9 months in her first pregnancy and 12 months in her second pregnancy and there was no back pain in her previous pregnancies. Biochemical examinations and erythrocyte sedimentation rate were normal. Vitamin D level was 31 ng/ml. There was no secondary cause of osteoporosis. Thoracolumbosacral orthosis (TLSO) was recommended to the patient by the neurosurgeon. BMD was measured with DXA. Lumbar total Z score −3.7, lumbar BMD = 0.687 g/cm^2^, femur total Z score −1.5, femur total BMD = 0.815 g/cm^2^ were detected. We started conservative treatment (cessation of breastfeeding, 1000 mg elemental calcium and 880 IU vitamin D) for the patient. The patient was admitted again with persistent severe back pain (VAS = 9/10) after six months. There was not a new fracture on MRI but lumbar total BMD was 0.690 g/cm^2^ (0.4% increase compared to baseline). Due to the lack of significant increase in BMD and persistent severe pain, 20 μg/day Teriparatide was added to her conservative treatment. Back pain significantly resolved in the second month of treatment (VAS = 2/10). Lumbar total BMD was 0.815 g/cm^2^ (18.1% increase compared to baseline) and femur total BMD was 0.826 g/cm^2^ (9.1% increase compared to baseline) (Table 1). Back pain completely resolved (VAS = 0/10), and teriparatide was discontinued at the 12th month. It was continued only with conservative treatment.

**Table 1:** Bone mineral density change before and after treatment.

	Postpartum 2th month	Postpartum 6th month	BMD change with conservative treatment (%)	Postpartum 18th month	BMD change after 12 months teriparatide (%)
Lumbar 1–4 BMD (g/cm^2^)	0.687	0.690	+0.4%	0.815	+18.1%
Femoral total BMD (g/cm^2^)	0.815	0.757	−7.1%	0.826	+9.1%

BMD: bone mineral density.

## 3 Discussion

If total body calcium reserve of a 55 kg mother is accepted as approximately 1000 g; Approximately 3% of the mother's calcium (30 g) reserve passes to the fetus during pregnancy. In other words, a full-term fetal skeleton contains 30 g of calcium, 20 g of phosphorus and 0.8 g of magnesium. Calcium transfer rate increases as the week of gestation progresses. While it is 60 mg/day to 24th week of pregnancy, it increases up to 300–350 mg/day after 36th week of pregnancy. The mother's calcium need increases during pregnancy. In addition to calcium transfer to the fetus, increased urinary calcium excretion during pregnancy also plays a role in this increase. While intestinal calcium absorption rate is 25% in a healthy adult, this rate increases approximately 2 times with the effect of increased calcitriol in pregnancy (Kovacs, 2016). Rapid mineralization of the fetal skeleton towards the end of pregnancy and calcium and vitamin D deficiencies in the diet lead to the resorption of maternal skeleton to meet the increasing calcium demand (Kovacs, 2001). Parathormone related peptide (PTHrP) is secreted from the placenta and breast tissue and increases bone turnover towards the end of pregnancy (Kovacs and Ralston, 2015; Black et al., 2000). DXA studies performed before and after pregnancy showed that the whole body and vertebral bone mineral density (BMD) decreased by 3–5%. Trabecular bone loss is more pronounced (Kovacs, 2011). Bone loss was observed in the third trimester in BMD, which was examined by heel ultrasonography (Gambacciani et al., 1995). During lactation (~15 months) approximately 4% of the mother's calcium reserve (about 40 g of calcium) passes to the baby (Kalkwarf and Specker, 2002). Increasing calcium demand in lactation is mainly met by bone resorption. In addition, urinary calcium excretion decreased in lactation differently from pregnancy (Kovacs, 2016). In lactation, both resorption and formation markers increase, but the increase in resorption markers is higher and net bone loss is observed (Sowers et al., 1995). Bone loss in lactation is higher than in pregnancy. There is a 3–10% reduction in BMD in lactation. Bone loss in lactation is associated with the duration of lactation and amenorrhea, and cannot be prevented by calcium supplementation. With the cessation of lactation and the start of menstruation again, BMD reaches its values in the early postpartum period within 6–18 months (Kovacs, 2011).

PLO is usually seen in the first pregnancy (70% primipar). The etiology is uncertain and its pathogenesis has been partially explained. Brain, breast and bone axis (high prolactin, low estradiol, high PTHrP) is thought to be responsible. Weight-bearing and lordotic pregnancy posture and pregnancy-induced immobilization (bed rest or hospitalization) are also considered in etiology. Genetic predisposition (a history of osteoporotic fracture in family members) and decreased physical activity in the peripubertal period may also be a risk factor. (Hadji et al., 2017; Laroche et al., 2017; Kyvernitakis et al., 2018; Zarattini et al., 2014).

To the best of our knowledge, the largest series in the literature on PLO are 102 cases of Hadji et al. and 52 cases of Laroche et al. (Hadji et al., 2017; Laroche et al., 2017). In the series of Hadji et al., the mean age was 34.1, the mean body mass index (BMI) was 22.7 kg/m^2^ and the mean number of vertebral fracture was 3.3/patient. The most common fractures were observed in the thoracolumbar region (most commonly T12 and L1). It has been reported that patients with PLO have significantly less physical activity in the peri-pubertal period than in the control group, and pregnancy-related diseases such as premature contractions, bleeding and hypertension are more common in patients with PLO (Hadji et al., 2017). In an average 6-year follow-up, 28% of patients with a history of PLO became pregnant again, and 20% of them had a PLO related fracture again (Kyvernitakis et al., 2018). In 52 cases of Laroche et al. PLO developed in 67% of cases in the first pregnancy, 15% in the second pregnancy and 10% in the third pregnancy. In 38% of PLO cases, the family had a history of osteoporotic fracture, while 27% had no risk factor for osteoporosis.

Our case is a rare case of PLO because, she was diagnosed after her third pregnancy and she has no history of PLO in her first two pregnancies.

The goals in PLO treatment should be to prevent the development of new vertebral fractures, to relieve the back pain and to increase BMD. Treatment options are in two forms, conservative treatment approach and specific pharmacological treatments (Kovacs and Ralston, 2015). In conservative treatment approach, lactation cessation (weaning off breastfeeding) and elemental calcium (1200 mg/day)/vitamin D supplementation (25 OH D vit > 30 ng/ml) is essential. Early mobilization (avoiding long bed rest), avoiding heavy lifting, supporting vertebral corsets/TLSO are also important. Vertebroplasty can be applied in necessary cases (Hadji et al., 2017; Laroche et al., 2017; Kyvernitakis et al., 2018; Phillips et al., 2000).

There is no standard treatment protocol or common opinion regarding the management of cases that do not respond to conservative treatment. There are no randomized controlled trials on specific pharmacologic treatments. It is not known whether the improvement is spontaneous or the effect of specific treatments. Treatment should be planned individually, according to the patient's age, severity of the disease, and desire for a pregnancy again.

In the literature, there are case reports using different pharmacological treatments such as bisphosphonates, teriparatide, calcitonin, denosumab, strontium ranelate in PLO treatment (Hadji et al., 2017; Laroche et al., 2017; Zarattini et al., 2014; Sánchez et al., 2016; Choe et al., 2012; Polat et al., 2015; Ozdemir et al., 2015; Tuna et al., 2019; O'Sullivan et al., 2006; Tanriover et al., 2009; Adamidou et al., 2012; Hong and Rhee, 2019). In the series (n = 52) of Larosche et al. 35% of patients received only conservative treatment, 62% received both conservative and specific pharmacological treatment (17% bisphosphonate, 21% teriparatide, 4% strontium ranelate and 4% calcitonin). Supportive vertebral corset was used in 21% of patients. Vertebroplasty was performed only in one patient. Annual lumbar BMD increase; 7% in the group receiving only conservative treatment, 10% in the group receiving bisphosphonate and 15% in the group receiving teriparatide (Laroche et al., 2017). Bisphosphonates remain in the bone matrix for years and the half-life in the bone is 10 years. Bisphosphonates pass through the placenta and have teratogenic effects on the fetus. Therefore, bisphosphonates may be risky for subsequent pregnancies of PLO patients who are in the reproductive period. Diarrhea, venous thromboembolism and serious skin reactions risks should be kept in mind in patients using strontium ranelate. Denosumab is a human monoclonal antibody developed against receptor activator nuclear factor kappa ligand (RANKL). In the publication of Sanches et al., it was stated that there was an increase of 17% in radius trabecular volume and 21% in trabecular thickness in the first year of denosumab treatment (Sánchez et al., 2016). It should be kept in mind that when denosumab is discontinued, if another antiresorptive treatment is not used, bone loss will restart rapidly and therefore it is recommended to continue with bisphosphonate treatment after denosumab. Teriparatide is an osteo-anabolic agent and does not accumulate in the bone. It does not pose any risk to the fetus in pregnancies that will occur after quitting teriparatide. Thus, teriparatide may be an option to add to conservative treatment in PLO patients with multiple vertebral fractures. Choe et al. stated that, there was a 19% increase in lumbar BMD and a 13% increase in femoral neck BMD after 18 months of teriparatide treatment in two PLO patients (Choe et al., 2012). Polat et al. reported that, the patient's symptoms related to pain improved in the second month of teriparatide treatment and after 18 months of treatment, there was a 27% increase in lumbar BMD (Polat et al., 2015). Hong et al. reported a meta-analysis of 30 studies on the effect of pharmacological treatment against PLO. They reported that significantly higher BMD change was observed in bisphosphonate or teriparatide compared to other and conservative treatments (bisphosphonate 18.2%; teriparatide, 17.0%; other 8.6%, conservative 7.9%) (Hong and Rhee, 2019).

In our patient, after the diagnosis of PLO, she was followed up with conservative treatment for six months, but her symptoms did not regress and there was no positive improvement in her BMD. Thus, we added teriparatide to her treatment. Symptomatic improvement began in the 2nd month and a significant increase (18%) in BMD was observed in the 12th month of teriparatide treatment. It should be kept in mind that there is a probable bone cancer risk in rats after prolonged teriparatide treatment. Thus, the duration of use is limited to 18–24 months (Langdahl et al., 2009).

## 4 Conclusion

In pregnant or breastfeeding patients with back pain, PLO should keep in mind in differential diagnosis. PLO is usually self-limiting with conservative treatment (weaning off breastfeeding and calcium/vitamin D supplementation), but specific pharmacological treatments (bisphosphonates or teriparatide) can be used in selected cases. Teriparatide does not accumulate in the bone, contributes to symptomatic improvement and increase in BMD thus, it may be an option to add to conservative treatment in PLO. Nonetheless, it may be suggested to wait for a spontaneous recovery response with a conservative treatment of 12–18 months before pharmacological treatment begins.

## Informed consent

Written informed consent was obtained from the patient who participated in this case.

## Financial disclosure

The authors declared that this case has received no financial support.

## Transparency document

Transparency document. Image 1

## Declaration of competing interest

The authors solemnly declare that there is not any conflict of interest in this manuscript.
